# Bambara–wheat composite flour: rheological behavior of dough and functionality in bread

**DOI:** 10.1002/fsn3.356

**Published:** 2016-03-04

**Authors:** Ochuko L. Erukainure, Jane N.C. Okafor, Akinyele Ogunji, Happiness Ukazu, Ebele N. Okafor, Ijeoma L. Eboagwu

**Affiliations:** ^1^ Department of Food TechnologyFederal Institute of Industrial ResearchOshodiLagosNigeria; ^2^ Department of Food TechnologyYaba College of TechnologyYabaLagosNigeria

**Keywords:** bambara nuts, composite flour, Mixolab, rheological behavior

## Abstract

The rheological behavior and functional properties of doughs from bambara–wheat composite flour was investigated. Bambara–wheat composite flour was prepared by substituting wheat with 0%, 10%, 15%, and 20% of bambara flour. The rheological behavior of their dough was analyzed with Mixolab. Breads produced from the flour were analyzed for physical characteristics. Organoleptic analysis was carried out by 20 panelists. Mixolab analysis revealed, except for stability time, depreciating values for dough consistency (C1), protein weakening (C2), starch gelatinization (C3), amylase activity (C4), and retrogradation (C5) as the inclusion of bambara flour increased. Physical characteristics of the loaves revealed significant (*P* < 0.05) decreasing bread volume and increasing specific volume, respectively, as bambara inclusion increased. There was significant (*P* < 0.05) difference between wheat bread and the bambara–wheat composites in all the studied quality attributes. 15% bambara–wheat composite bread was the most accepted amongst the composite breads. Inclusion of bambara flour improved the protein behavior of the composite, but did not evidently show benefits in the baking characteristics.

## Introduction

Over the years there have been increasing demands for convenience in consumed food by consumers. Amongst such foods, the demand for bread ranks the highest. This has been attributed to its low price, nutritive value, and simplicity of production (Martín et al. 2007). Bread refers to a perishable food prepared by baking a dough of wheat flour, water and salt, fermented by specific microorganisms particularly *Saccharomyces cerevisiae* (Ávila et al. 2007). It is regarded as one of the oldest staples, and a major source of carbohydrates in the human diet. Due to the low lysine content, it is often described as a nonbalanced diet (Agu et al. 2010; Okafor et al. 2012).

There have been increased interests in wheat flour supplementation, especially with legumes in the production of composite flours. This has been attributed to improved essential amino acid balance of baked food products (Kadam et al. 2012). Among such legumes is the bambara nut (*Vigna subterranea*).

Bambara nuts are grain legumes indigenous to sub‐Saharan Africa. Though an underutilized crop, it is ranked next to cowpea (Barimalaa et al. 2005) and has been described a complete food owing to its nutritional composition of an average 63% carbohydrate, 19% protein and 6.5% fat, thus making it a major source of dietary protein (Food and Agricultural Organization of the United States (FAO), 2005). They are also good sources of fiber, calcium, iron and potassium (Hillocks et al. 2012). Abdualrahman et al. (2012) reported increased protein content with a concomitant decrease of carbohydrate contents in bambara–wheat bread, thus indicating an improved nutrition value in bread.

To the best of our knowledge, this is the first time the rheological properties of bambara‐supplemented wheat flour are being ascertained with Mixolab. This paper therefore aims at reporting the rheological behavior of bread dough from wheat flour supplemented with different percentage of bambara nut flour, using standard Chopin+ protocol by Mixolab.

## Materials and Methods

### Plant materials

Wheat flour and bambara nuts were purchased from a local market at Oyingbo, Lagos, Nigeria. Clean bambara seeds were carefully sorted, decorticated with a hammer mill and then blended to fine flour. Both flours were sieved and stored in airtight containers until further analysis.

Composite flours were produced by substituting wheat flour with bambara flour at 0%, 10%, 15%, and 20%, respectively, and were coded as ZON, BWA, YBI, and PCB.

### Determination of rheological behavior and functionality of dough

Rheological behavior and functional properties of doughs of the developed composite flours were determined by Mixolab analysis using standard Chopin+ protocol according to the manufacturer's manual, which consisted of a heating/cooling cycle after a mixing time at a constant mixing speed of 80 rpm. The analyses were carried out at constant water absorption; the required amount of flour for analysis was calculated by Mixolab^®^ software according to input values of flour mixtures moisture as well as water absorption (Pastukhov and Dogan 2014).

### Production of wheat and bambara–wheat composite breads

Breads were produced from the wheat and composite flours, using standard bread baking procedures as described by Pyke (1976) and modified by the Federal Institute of Industrial Research, Oshodi (FIIRO), Lagos, Nigeria. The recipes and formula are depicted in Table 1. The amount of water needed to make the dough was estimated from the water absorption by Mixolab analysis.

**Table 1:** Recipes for production of wheat and bambara–wheat composite breads

Recipes	Weight (g)(%)
Flour	87
Salt	2
Sugar	7
Fat	3
Yeast	1

### Physical characteristics of bread loafs

The loaf volume was determined immediately after baking by the rape seed displacement method (Standard Organization of Nigeria (SON), 1976). Specific volume was determined from the weight and volume data.

### Organoleptic analysis of baked breads

The bread products were subjected to sensory evaluation by a total of 20 semi‐trained panelists drawn from the Staff of Federal Institute of Industrial Research, Oshodi‐Lagos, Nigeria. The selected participants were familiar with the bread. The samples were coded and slices were served in clean white plates to the panelist in a sensory evaluation booth with fluorescent lights on. The panelists were asked to eat and evaluate the attributes: appearance/loaf shape, crust color, crumb color, crumb texture, taste, chew ability, aroma, and overall acceptability, using a 9‐point Hedonic scale where 1 = extremely unacceptable and 9 = extremely acceptable (Okafor et al. 2012).

### Statistical analysis

All samples were done in triplicate and repeated at least thrice for liability of data. Statistical significance was established using one‐way analysis of variance (ANOVA), and data were reported as mean ± standard deviation. Significant difference was established at *P* < 0.05. Statistical analyses were carried out, using SPSS for Windows, version 15.0 (SPSS Inc., Chicago, IL).

## Results and Discussion

### Rheological behavior of composites dough

Mixolab measures the rheological properties of the dough subjected to the dual stress of mixing and temperature change between two mixing blades absorption (Pastukhov and Dogan 2014). The curve pattern of the Mixolab result also gives a quality indication of the flour sample. There are five distinct phases on the Mixolab curve C1–C5 each measuring different rheological parameters of the flour.

**Figure 1:**
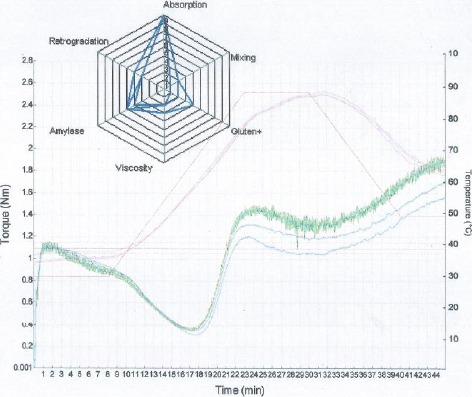
Mixolab^®^ curves comparing the rheological behaviors of bambara–wheat doughs to whole wheat flour dough.

**Figure 2:**
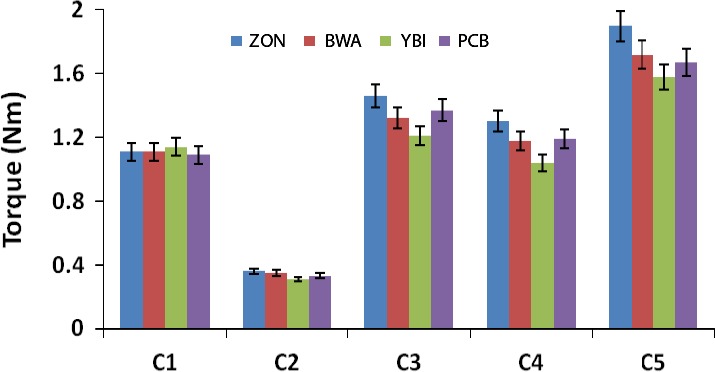
Values for Mixolab curve (C1–C5) of wheat and bambara–wheat composite doughs. Values = mean ±SD; *n* = 3.

C1 represents the highest point on the curve, and it records dough development and stability time, respectively. It also measures protein behavior of doughs. In this study, a gradual decrease in dough development time was observed for the test samples with increasing substitution with bambara flour as shown in Figures 1, 2. There was also a noticeable increase in the stability time from 4.58 min in whole wheat flour to 6.68 min at 20% substitution. The observed longer stability with increased bambara flour can be attributed to the strength of protein molecules of the composite flours. Okpuzor et al. (Okpuzor et al. 2010) identified ten and twelve different types of proteins in malted and dry seeds of bambara nuts, which may explain the observed stability time.

**Figure 3:**
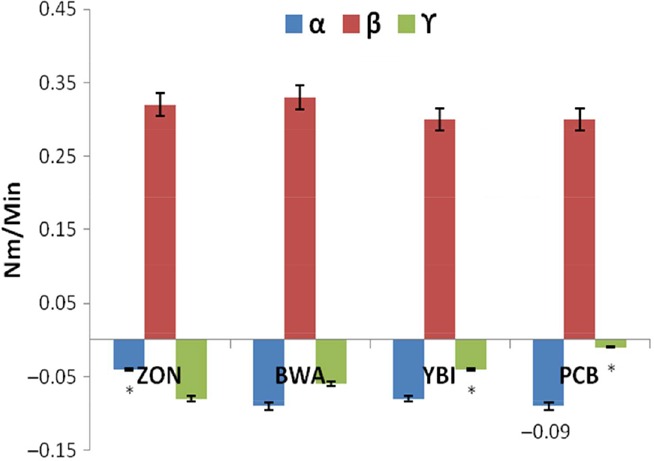
*α*,* β* and γ slopes of Mixolab curves of wheat and bambara–wheat composite doughs. Values = mean ±SD; *n* = 3. *Statistically significant at *P* < 0.05.

C2 portrays protein weakening of dough as a function of mechanical work and temperature (Pastukhov and Dogan 2014). Heating causes a breakage of the protein links as well as denaturing. A high value of C2 indicates good protein quality (Aprodu et al. 2010). In this study, the values were observed to depreciate with concomitant rise in dough temperature as bambara flour inclusion increased (Figs. 1, 2). This observation corresponds to previous studies by Collar et al. (2007), who reported that good protein exhibits great stability during heating, but later becomes weakened and reduced, and slowly breaks down. The observed protein weakening of the dough is further portrayed by values of the *α* slopes as seen in Figure 3, which indicates protein weakening speed influenced by heat (Pastukhov and Dogan 2014).

**Figure 4:**
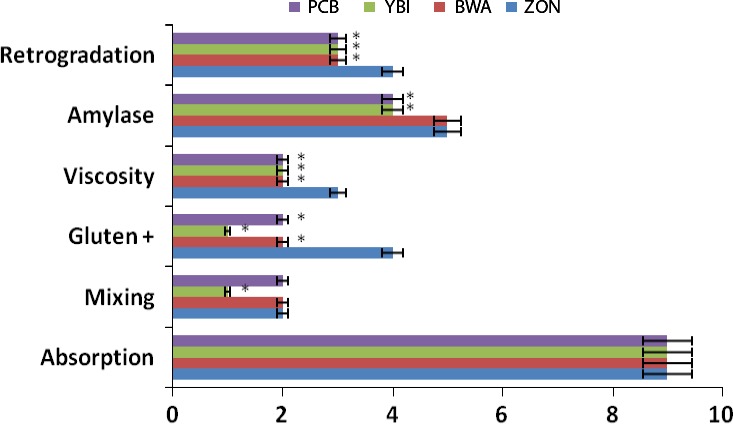
Effect of bambara inclusion on functional properties of bambara–wheat dough. Values = mean ±SD; *n* = 3. *Statistically significant at *P* < 0.05.

The torque at C3 reduced from 1.46 Nm in whole wheat flour to 1.32 Nm and 1.21 Nm in 10% and 20% of bambara flour inclusion, respectively (Figs. 1, 2), implying a reduction in starch gelatinization as portrayed by the *β* slope values (Fig. 3). This point in the Mixolab graph (C3) indicates maximum viscosity and has been attributed to quick rupture of starch granules, leading to lower pasting temperatures and to higher paste consistency (Pastukhov and Dogan 2014). The increased dough temperature together with water liberated by the denatured proteins (C2) causes the starch granules to swell and burst, thereby inducing an increased dough consistency (Aprodu et al. 2010). This may explain the observed reduced viscosity index on increasing bambara flour inclusion as depicted in Figure 4.

As the temperature of the dough increases near peak (C4), a decrease in the rate of their consistency is observed as indicted by the *γ* slope in Figure 3 (Aprodu et al. 2010). At this point, amylose and amylopectin are free catch water leading to an increased amylase activity, thereby decreasing viscosity. There was a steady decline in the amylase index values with increased bambara flour inclusion, depicting a higher amylase activity in 20% inclusion as compared to 0% and 10% inclusions (Figs. 1, 2). This indicates that inclusion of bambara flour increased the ability of the starch to withstand amylolysis.

A higher value of C5 portrays a longer shelf stability of the finished product (Dhaka et al. 2012). Formation of gel has been shown to cause bread deterioration. Upon cooling, gelatinized amylose molecules begin to recrystallize, leading to separation of liquid from the gel. As the inclusion of bambara flour increased, there was a decrease in the C5 torque value indicating a decrease in the retrogradation time (Figs. 1, 2), thus implying that products from 20% and 10% composite flour dough will be slightly shelf stable than the wheat flour product.

Based on these results, the mechanism by which bambara nuts influences the dough behavior of wheat can be summarized as thus: the reported protein content of bambara nuts increased the formation and stability time of the bambara flour substituted wheat. As the mixing temperature increases, there is an increase in conformational entropy of these proteins coupled with a hydrophobic effect causing an unfolding of the polypeptide chain and finally denaturing of the proteins as characterized by the weakened dough. The water molecules released due to the hydrophobic effect is absorbed in the amorphous space of the flour starch and binds tightly to the double helical structures of amylopectin. This causes swelling and leaching of soluble amylose molecules, and finally the granule structure disintegrates. As the temperature drops, there is a decrease in endothermic enthalpy leading to retrogradation owing to hydrogen bonding between chains and the embedded water molecules.

### Physical characteristics of loaves

**Figure 5:**
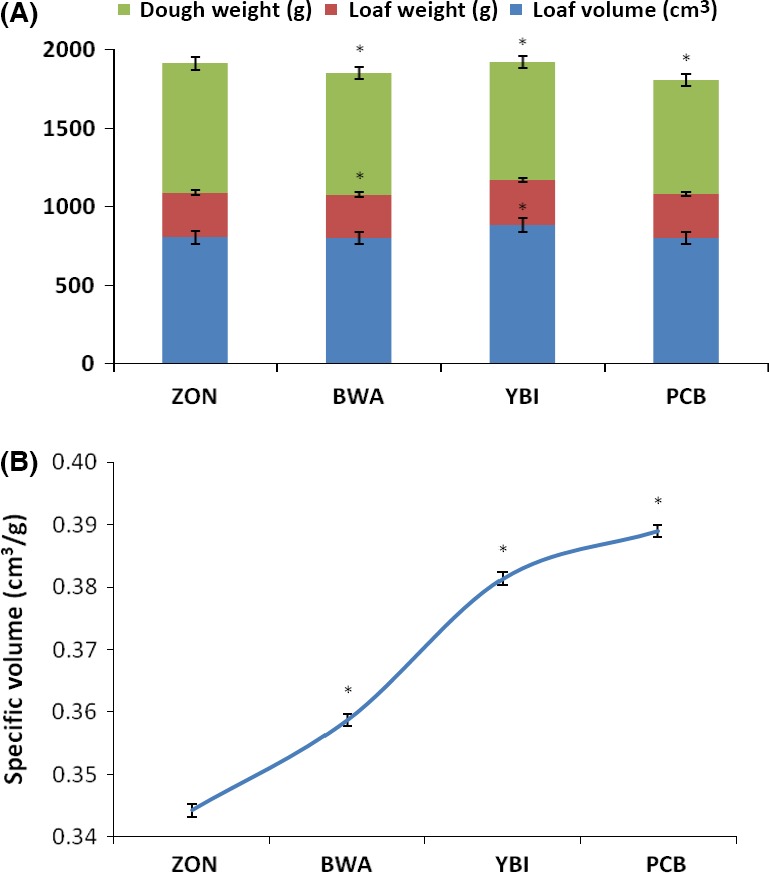
Physical characteristics of bambara–wheat breads. Value = mean ±SD; *n* = 3. *Statistically significant at *P* < 0.05. (A) Dough weight, loaf weight, and loaf volume of bambara–wheat breads. (B) Specifi c volume of bambara–wheat breads.

The weight of the doughs after mixing is depicted in Figure 5A. Significant (*P* < 0.05) higher dough weight was observed on 15% inclusion of bambara flour, while there was no significant difference amongst the other doughs. This was, however, not evident in the loaf weights as no significant difference was observed.

The loaf volume was observed to significantly (*P* < 0.05) decrease with increasing bambara inclusion. This is similar to previous reports by Okafor et al. (2012) and Agu et al. (2010) who reported decreased loaf volume on substitution with mushroom and fluted pumpkin flour, respectively. However, an increased specific volume was observed with increasing bambara inclusion (Fig. 5B). This can be attributed to the weight and volumes of the bread loaves as the specific volume are a function of both. This observation contrasts that of Okafor et al. (2012) who reported a rather decreased specific volume on wheat substitution. Kamaljit et al. (2011) reported an increased specific volume with increase in oat flour inclusion. The observed increased specific volume can be attributed to reduced gluten content as is evident by C3 in the Mixolab curves (Figs. 1, 2).

### Organoleptic attributes

**Figure 6:**
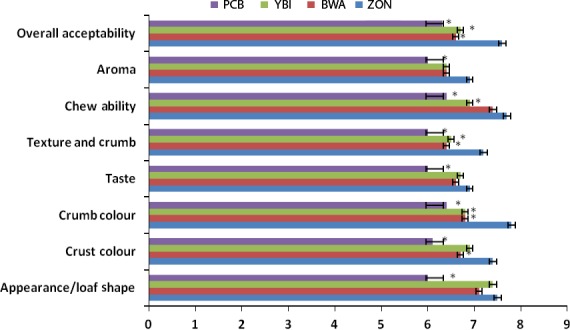
Organoleptic properties of bambara–wheat breads. Value = mean ±SD; *n* = 20. *Statistically significant at *P* < 0.05

Sensory characteristics of food particularly taste and flavor has been shown to significantly influence consumers' choice of food (Erukainure et al. 2013). Psychological factors such as personality, mood and experience, and attitudes like sensory properties, health/nutrition, and price/value have been identified as major determinants of food choice. In this study, the breads were presented to 20 panelists to evaluate the selected quality attributes. There was significant (*P* < 0.05) difference between wheat bread and the composite breads in all the studied quality attributes as shown in Figure 6. Here the 15% bambara–wheat composite bread was observed to be the most accepted amongst the composite breads. Generally, the bambara flour gave the bread a unique taste, color, and texture which increased with increasing inclusion. Familiarity with the products may also influence the panelists' preference as training and experience on the sensory method of profiling has been argued to increase sensory ability (Hughson and Boakes 2002; Labbe et al. 2004; Bitnes et al. 2007).

## Conclusion

Inclusion of bambara flour improved the protein behavior of the composite, but did not evidently show benefits in the baking characteristics. The improved protein behavior can be attributed to the protein quality of Bambara nuts, thus ascertaining the influence of the proteins on the rheological properties of the composite flour. However, there is need to study the storage and loss moduli of the composites in order to ascertain the energy stored and dissipated, respectively.

## Conflict of Interest

The authors declare no conflict of interest.
